# Supplementary material for: PfCRT mutations conferring piperaquine resistance in falciparum malaria shape the kinetics of quinoline drug binding and transport
Source: PLoS Pathog. 2023 Jun 7;19(6):e1011436. doi: 10.1371/journal.ppat.1011436 (PMC10281575; doi:10.1371/journal.ppat.1011436)
Supplement: S1 Table — (PDF) [file ppat.1011436.s001.pdf]

S1 Table. Glide SP docking scores (kcal/mol) of the 10 poses generated with the IFD protocol for the binding of CQ or PPQ in the cavity of PfCRT^{Dd2}, PfCRT^{7G8}, PfCRT^{Dd2_F145I}, PfCRT^{Dd2_H97Y}, PfCRT^{Dd2_G353V}, PfCRT^{Dd2_M343L} and PfCRT^{Dd2_H97Y_F145I}.

PfCRT^{Dd2}

#CQ	Docking score	#PPQ	Docking score
1	-6.743	1	-9.329
2	-6.562	2	-8.647
3	-5.734	3	-8.550
4	-5.729	4	-7.941
5	-5.684	5	-7.888
6	-5.640	6	-6.924
7	-5.428	7	-6.895
8	-5.168	8	-6.697
9	-4.713	9	-6.496
10	-4.324	10	n.a.

PfCRT^{7G8}

#CQ	Docking score	#PPQ	Docking score
1	-6.287	1	-9.606
2	-5.874	2	-9.077
3	-5.874	3	-8.631
4	-5.785	4	-8.469
5	-5.566	5	-8.298
6	-5.540	6	-7.927
7	-5.472	7	-7.504
8	-5.095	8	-7.366
9	n.a	9	-7.043
10	n.a	10	-6.750

PfCRT^{Dd2_F145I}

#CQ	Docking score	#PPQ	Docking score
1	-6.884	1	-8.990
2	-6.884	2	-8.400
3	-6.663	3	-8.107
4	-5.764	4	-7.969
5	-5.575	5	-7.796
6	-5.383	6	-7.683
7	-5.193	7	-7.456
8	-5.086	8	-7.200
9	-5.075	9	-7.146
10	-3.846	10	-6.758

PfCRT^{Dd2_H97Y}

#CQ	Docking score	#PPQ	Docking score
1	-6.663	1	-9.151
2	-6.289	2	-8.838
3	-5.715	3	-8.230
4	-5.543	4	-7.944
5	-5.524	5	-7.858
6	-5.091	6	-7.757
7	-5.063	7	-7.119
8	-4.956	8	-6.677
9	-4.651	9	n.a
10	n.a	10	n.a

PfCRT^{Dd2_G353V}

#CQ	Docking score	#PPQ	Docking score
1	-6.670	1	-9.086
2	-6.583	2	-8.645
3	-5.960	3	-8.154
4	-5.587	4	-7.732
5	-5.552	5	-7.585
6	-5.526	6	-7.067
7	-5.501	7	-6.497
8	-5.317	8	n.a
9	-4.612	9	n.a
10	n.a	10	n.a

PfCRT^{Dd2_M343L}

#CQ	Docking score	#PPQ	Docking score
1	-6.169	1	-8.350
2	-5.614	2	-8.012
3	-5.607	3	-7.786
4	-5.577	4	-7.741
5	-5.387	5	-7.733
6	-5.322	6	-7.655
7	-5.210	7	-6.940
8	-5.201	8	-6.885
9	-5.192	9	-6.800
10	-4.775	10	-6.584

PfCRT^{Dd2_H97Y_F145I}

#CQ	Docking score	#PPQ	Docking score
**1**	-6.617	**1**	-10.109
**2**	-6.303	**2**	-9.881
**3**	-5.590	**3**	-8.941
**4**	-5.516	**4**	-8.714
**5**	-5.353	**5**	-7.995
**6**	-5.316	**6**	-7.699
**7**	-5.268	**7**	-7.594
**8**	-5.059	**8**	-7.491
**9**	-4.858	**9**	n.a
**10**	-4.513	**10**	n.a
