# Supplementary material for: PfCRT mutations conferring piperaquine resistance in falciparum malaria shape the kinetics of quinoline drug binding and transport
Source: PLoS Pathog. 2023 Jun 7;19(6):e1011436. doi: 10.1371/journal.ppat.1011436 (PMC10281575; doi:10.1371/journal.ppat.1011436)
Supplement: S2 Table — (PDF) [file ppat.1011436.s002.pdf]

S2 Table. Primers used for the mutagenesis of *pfcrt* onto the codon-optimized *pfcrt*^{Dd2} sequence in the pSP64T vector backbone. The *pfcrt* sequence is shown underneath, with the globin UTR sequences in green, and the start and stop codons underlined.

N°	Name	Sequence (5' to 3')
**1**	5'globin-for	GCAGAAGCTCAGAATAAACG
**2**	3'globin-rev	GTAGCTTAGAGACTCCATTCG
**3**	H97Y-for	CCTCTGAAACCTATAACTTCATCTG
**4**	H97Y-rev	CAGATGAAGTTATAGGTTTCAGAGG
**5**	F145I-for	TATTTTGGCTATCATCGGTTTGAC
**6**	F145I-rev	TCAAACCGATGATAGCCAAAATAAC
**7**	M343L-for	TCTCCACTCTGACCTAC
**8**	M343L-rev	GTAGGTCAGAGTGGAGA
**9**	G353V-for	GCATTCAAGTTCCAGCTAC
**10**	G353V-rev	GTAGCTGGAACTTGAATGC
**11**	V141A-for	GCCTGCTCTGCTATTTTGGC
**12**	V141A-rev	GCCAAAATAGCAGAGCAGGC
**13**	S257A-for	CAATTATTCACCGCCTGCTTGA
**14**	S257A-rev	TCAAGCAGGCGGTGAATAATTG
**15**	I260A-for	CTCCTGCTTGGCTTTGCCAG
**16**	I260A-rev	CTGGCAAAGCCAAGCAGGAG

AATACAAGCTTGCTTGTTCTTTTTGCAGAAGCTCAGAATAAACGCTCAACTTTGGCAGATCCTCGA
GGGTACAAAATTGAAGATGAAGTTCGCCTCTAAGAAGAACAATCAAAAGAACTCCTCCAAGAATG
CTGAAAGAGCTAGAGCTGCTGATAATGCTGCTCAAGAAGGTAACGGTTCTAGATTGGGTGGTGGT
TCTTGTTTGGGTAAATGTGCTCATGCTGCTAAAGCTGCCTTCAAAGAAATCAAGGACAACATCTTC
ATCTACATCTTGTCCATCATCTACTTGTCCGTTTGCGTTATTGAAACCATCTTCGCCAAGAGAACC
TTGAACAAGATTGGTAACTACTCTTTCGTTACCTCTGAAACCCATAACTTCATCTGCATGATCATGT
TCTTCATCGTCTATTCCTTGTTCGGTAACAAGAAGGGTAACTCCAAAGAAAGACACAGATCCTTCA
ACTTGCAATTCTTCGCCATTTCTATGTTGGATGCCTGCTCTGTTATTTTGGCTTTCATCGGTTTGAC
TAGAACTACCGGTAACATCCAATCTTTCGTCTTGCAATTGTCCATTCCAATCAATATGTTCTTCTGC
TTCTTGATCTTGAGATACAGATACCACTTGTACAATTACTTGGGTGCCGTTATTATTGTCGTTACCA
TTGCCTTGGTTGAAATGAAGTTGTCCTTCGAAACCCAAGAAGAAAACTCCATCATCTTCAACTTGG
TTTTGATCTCCTCATTGATCCCAGTTTGTTTCTCTAACATGACCAGAGAAATCGTTTTCAAGAAGTA
CAAGATCGACATCTTGAGATTGAACGCTATGGTTTCCTTCTTCCAATTATTCACCTCCTGCTTGATT
TTGCCAGTTTACACCTTGCCATTCTTGAAAGAATTGCACTTGCCATACAACGAAATTTGGACCAAC
ATCAAGAATGGTTTCGCTTGTTTGTTCTTGGGTAGAAACACCGTTGTTGAAAACTGTGGTTTGGGT
ATGGCTAAGTTGTGTGATGATTGTGATGGTGCTTGGAAAACTTTCGCTTTGTTCTCCTTCTTCTCC
ATTTGCGATAACTTGATCACCTCCTACATTATCGATAAGTTCTCCACTATGACCTACACTATCGTAT
CTTGCATTCAAGGTCCAGCTACTGCTATTGCTTACTACTTCAAGTTCTTGGCTGGTGATGTTGTTA
TTGAACCTAGATTATTGGACTTCGTCACCTTGTTTGGTTACTTGTTCGGTTCCATTATCTACAGAGT
CGGTAACATCATCTTGGAAAGAAAGAAGATGAGAAACGAAGAAAACGCTGATTCTGCTGGTGCTT
TGACTAATGTTGATTCTGCTGCTACTCAACCTAGGTAATCCATGGGATCTGGTTACCACTAAACCA
GCCTCAAGAACACCCGAATGGAGTCTCTAAGCTACATAATACCAACTTACACTTTACAAAATGTTG
TCCCCCAAAATGTAGCCATTCGTATCT
