# Supplementary figures and images for: PfCRT mutations conferring piperaquine resistance in falciparum malaria shape the kinetics of quinoline drug binding and transport
Source: PLoS Pathog. 2023 Jun 7;19(6):e1011436. doi: 10.1371/journal.ppat.1011436 (PMC10281575; doi:10.1371/journal.ppat.1011436)

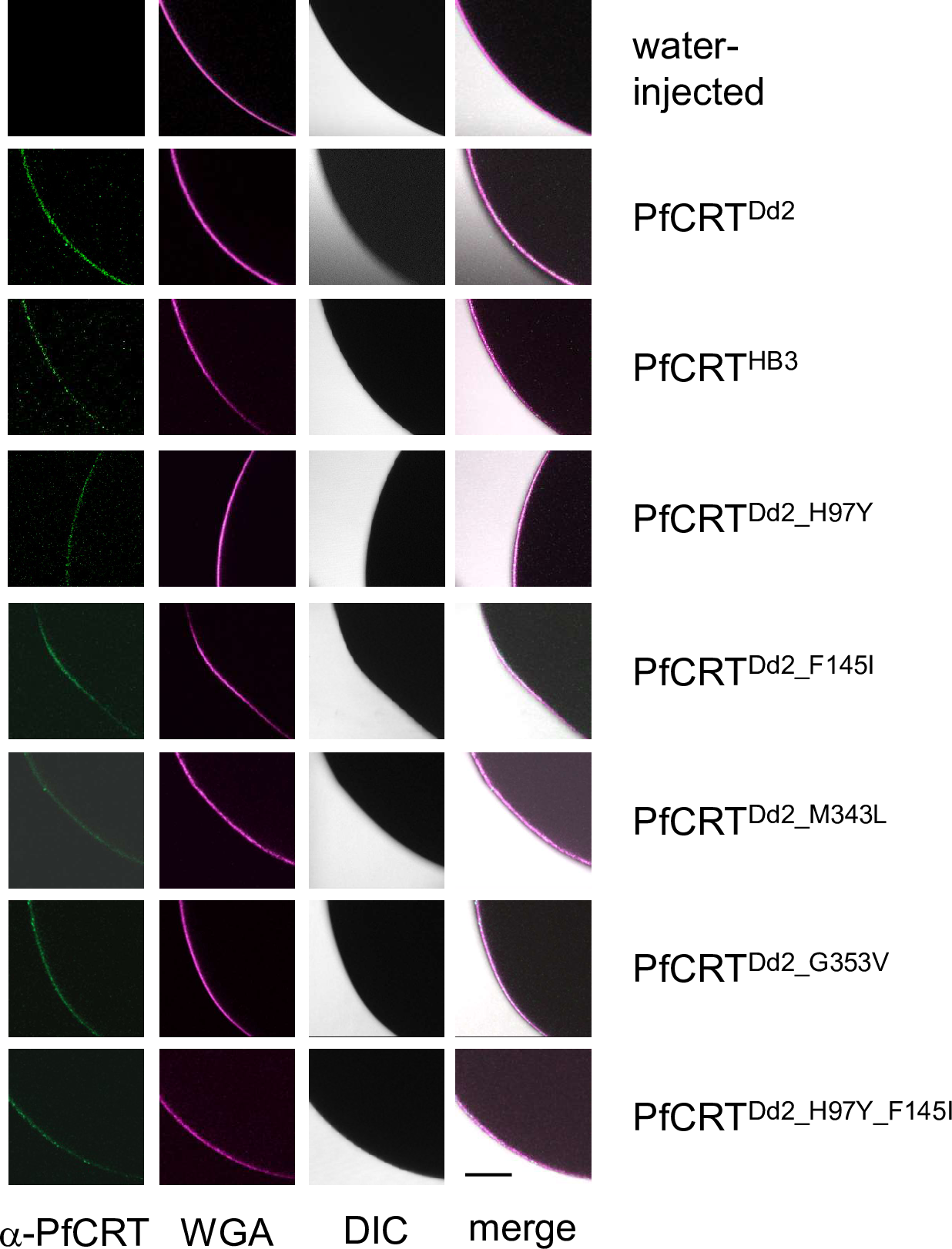

Supplement: S1 Fig — Immunofluorescence images of fixed PfCRT -expressing oocytes and water-injected control oocytes. First panel from the left, fluorescence image of PfCRT using a specific guinea pig antiserum primary antibody (α-PfCRT) and the Alexa Fluor 633 anti-guinea pig secondary antibody. Second panel, fluorescence image of wheat germ agglutinin (WGA) conjugated to Alexa Fluor 488. Third panel, differential interference contrast (DIC) image. Fourth panel, overlay. Scale bar, 135 μm. For visualization purposes, contrast was enhanced. (TIF) [file ppat.1011436.s004.tif]

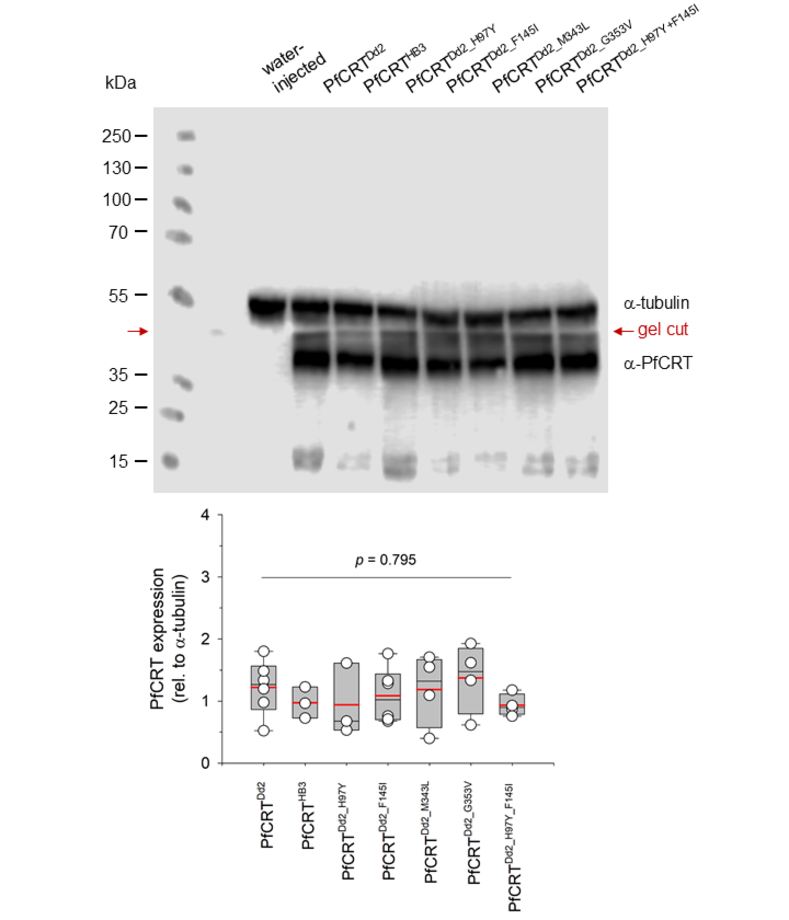

Supplement: S2 Fig — Western blot analyses of total lysates from oocytes expressing PfCRT variants and water-injected oocytes, using the polyclonal guinea pig antiserum specific to PfCRT and, as a loading control, mouse monoclonal anti-α-tubulin antibody. The luminescence signals from independent Western blot analyses were quantified, yielding the PfCRT expression levels relative to the internal standard α-tubulin. A box plot analysis is overlaid over the individual data points (independent biological replicates) with the median (black line), mean (red line), and 25 and 75% quartile ranges being shown. Statistical significance was assessed using the Brown-Forsythe ANOVA and found to be p = 0.795. (TIF) [file ppat.1011436.s005.tif]

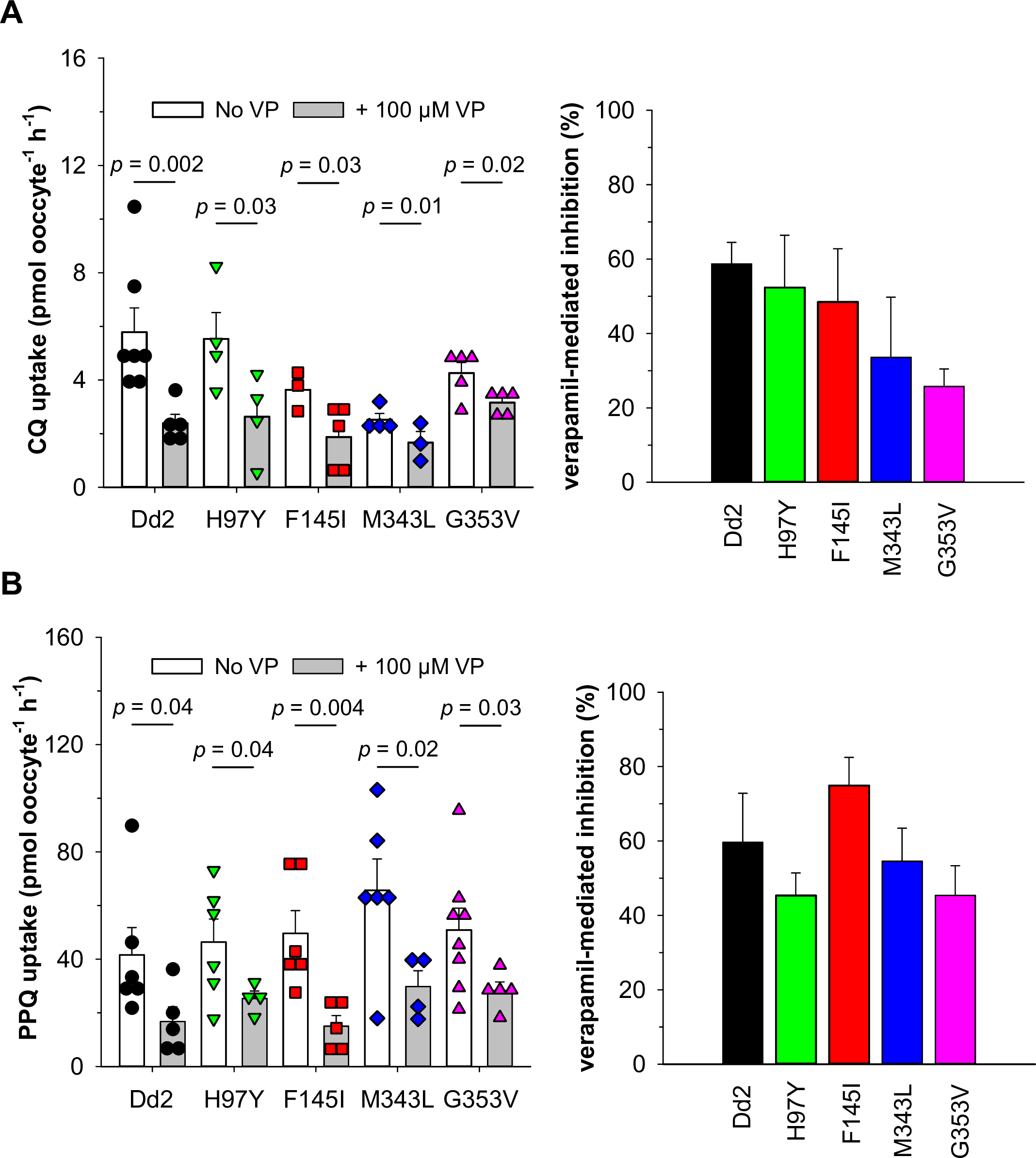

Supplement: S3 Fig — The inhibitory effect of adding 100 μM verapamil (VP) to an extracellular medium containing a total concentration of 50 μM A, chloroquine (CQ) or B, piperaquine (PPQ), was evaluated in water-injected (control) and oocytes expressing PfCRTDd2 (Dd2), PfCRTDd2_H97Y (H97Y), PfCRTDd2_F145I (F145I), PfCRTDd2_M343L (M343L) or PfCRTDd2_G353V (G353V). Left panels, data is shown as individual biological replicates (coloured symbols) overlaid onto bars representing the Mean ± S.E.M. (error bars). A one-tailed Student’s t-test was performed between untreated (no VP) and treated (+ 100 μM VP) samples, and the obtained p values are shown. Right panels, the uptake in VP-treated samples was divided by the uptake in their corresponding untreated pairs and subtracted from 100% to get the percentage of uptake inhibition mediated by the presence of 100 μM verapamil in the extracellular medium. Error bars are S.E. (TIF) [file ppat.1011436.s006.tif]

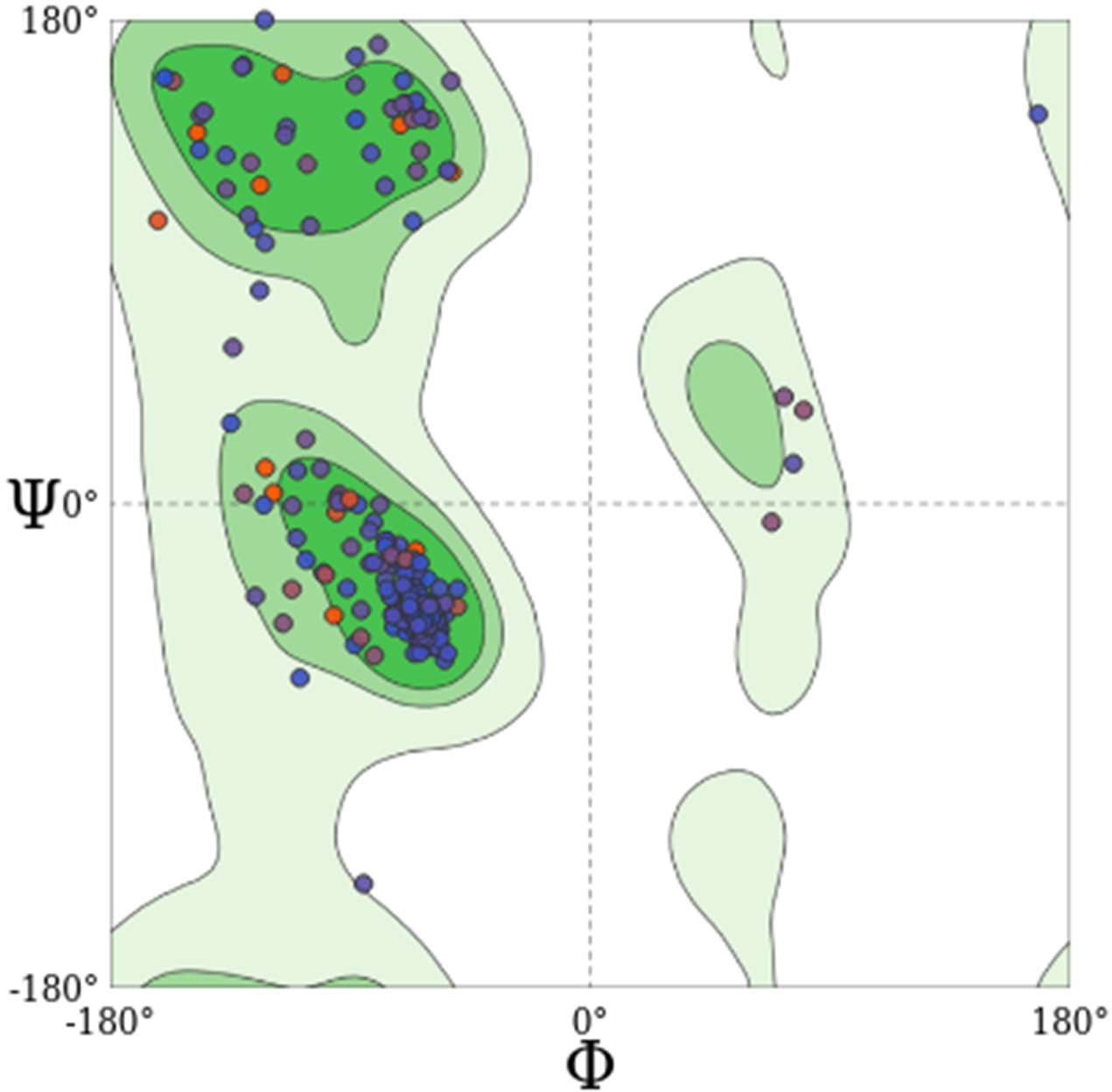

Supplement: S4 Fig — The structure validation was performed with MolProbity, implemented in the SWISS-MODEL server [34]. 96.64% of the residues are positioned in the favored regions and only 0.28% are in outlier regions. The residues, indicated by dots, are colored according to the QMEAN parameter, indicating the residue quality. The color scale goes from red (bad quality) to blue (good quality). (TIF) [file ppat.1011436.s007.tif]

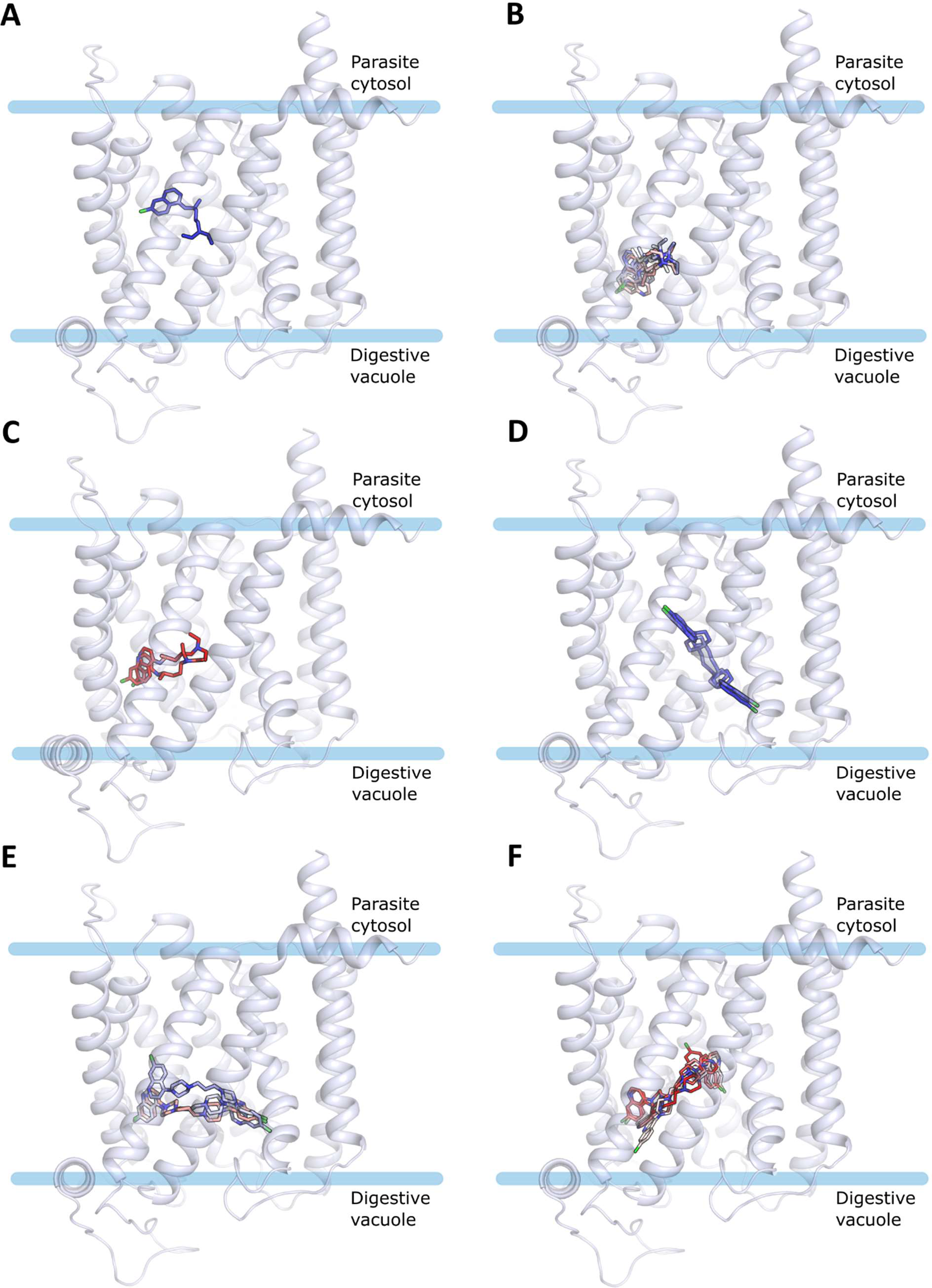

Supplement: S5 Fig — A blue-to-red color scale shows the generated docking poses for CQ (A-C) and PPQ (D-F), ranked from the best (blue) to the worst (red) docking score. (TIF) [file ppat.1011436.s008.tif]

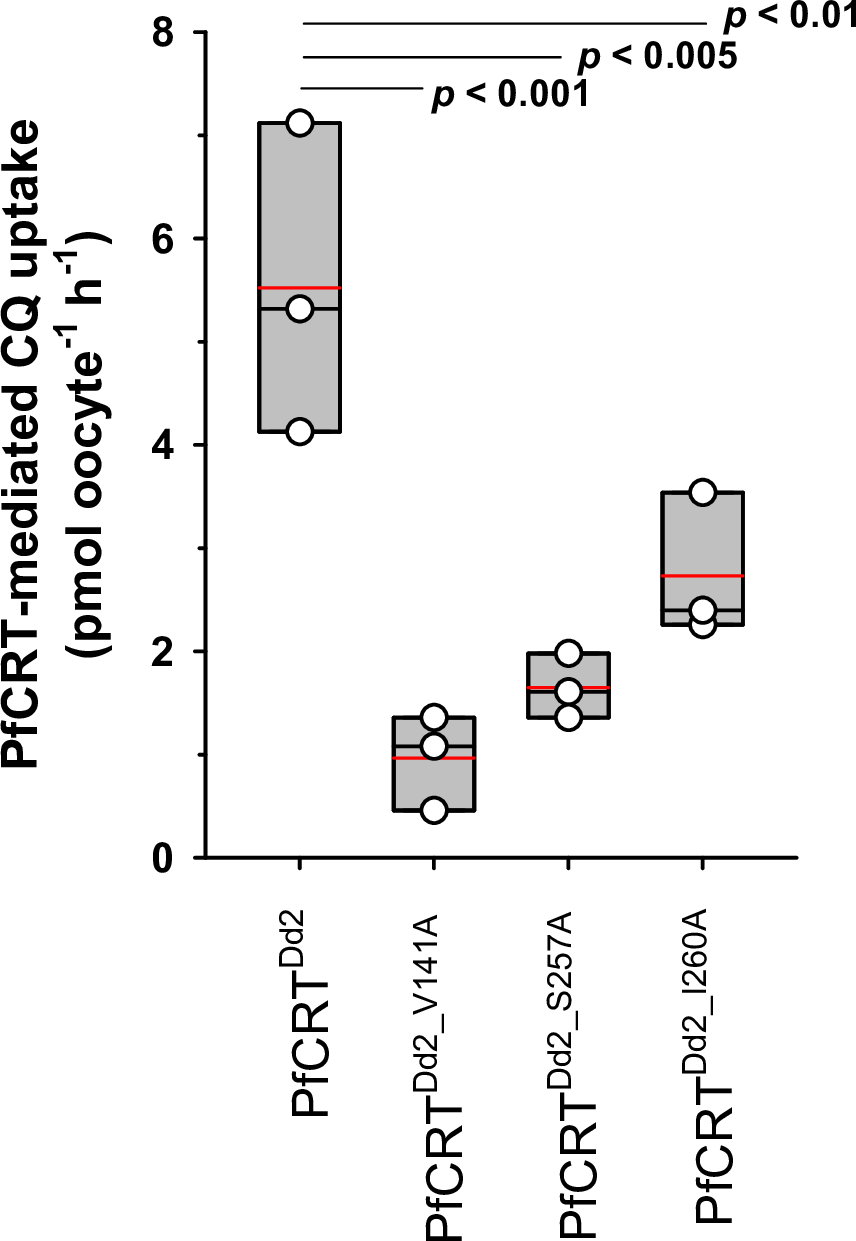

Supplement: S6 Fig — The PfCRT-mediated transport of CQ was measured in oocytes expressing PfCRTDd2, PfCRTDd2_V141A, PfCRTDd2_S257A or PfCRTDd2_I260A. A box plot analysis is overlaid over the individual data points (independent biological replicates) with the median (black line), mean (red line), and 25 and 75% quartile ranges being shown. Multiple comparisons versus the PfCRTDd2 control group was performed (Holm-Sidak ANOVA). (TIF) [file ppat.1011436.s009.tif]

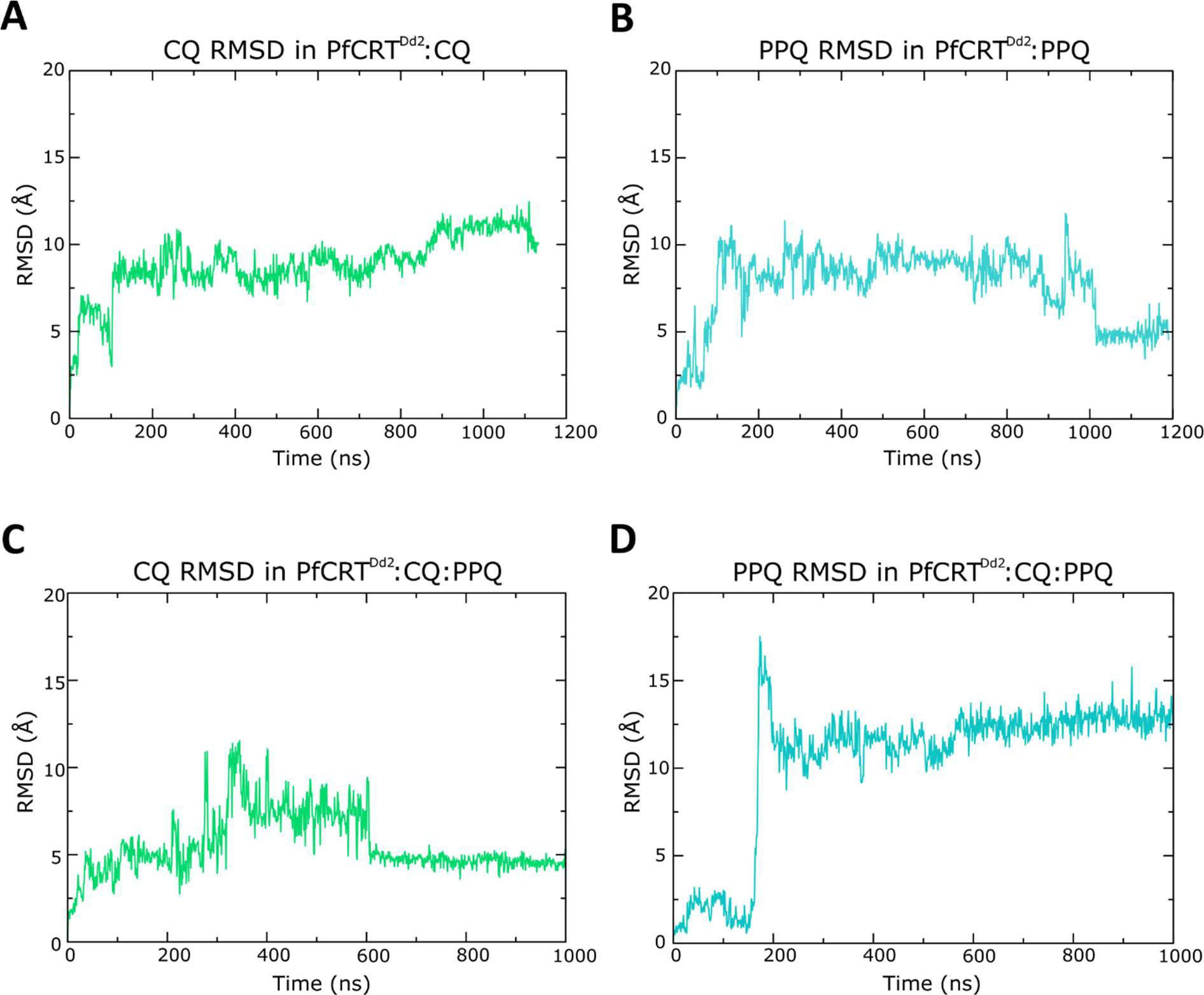

Supplement: S7 Fig — A, B. CQ (A) and PPQ (B) RMSD in their respective complexes with PfCRTDd2 (PfCRTDd2:CQ and PfCRTDd2:PPQ). C, D. CQ (C) and PPQ (D) RMSD in the PfCRTDd2:CQ:PPQ complex. (TIF) [file ppat.1011436.s010.tif]

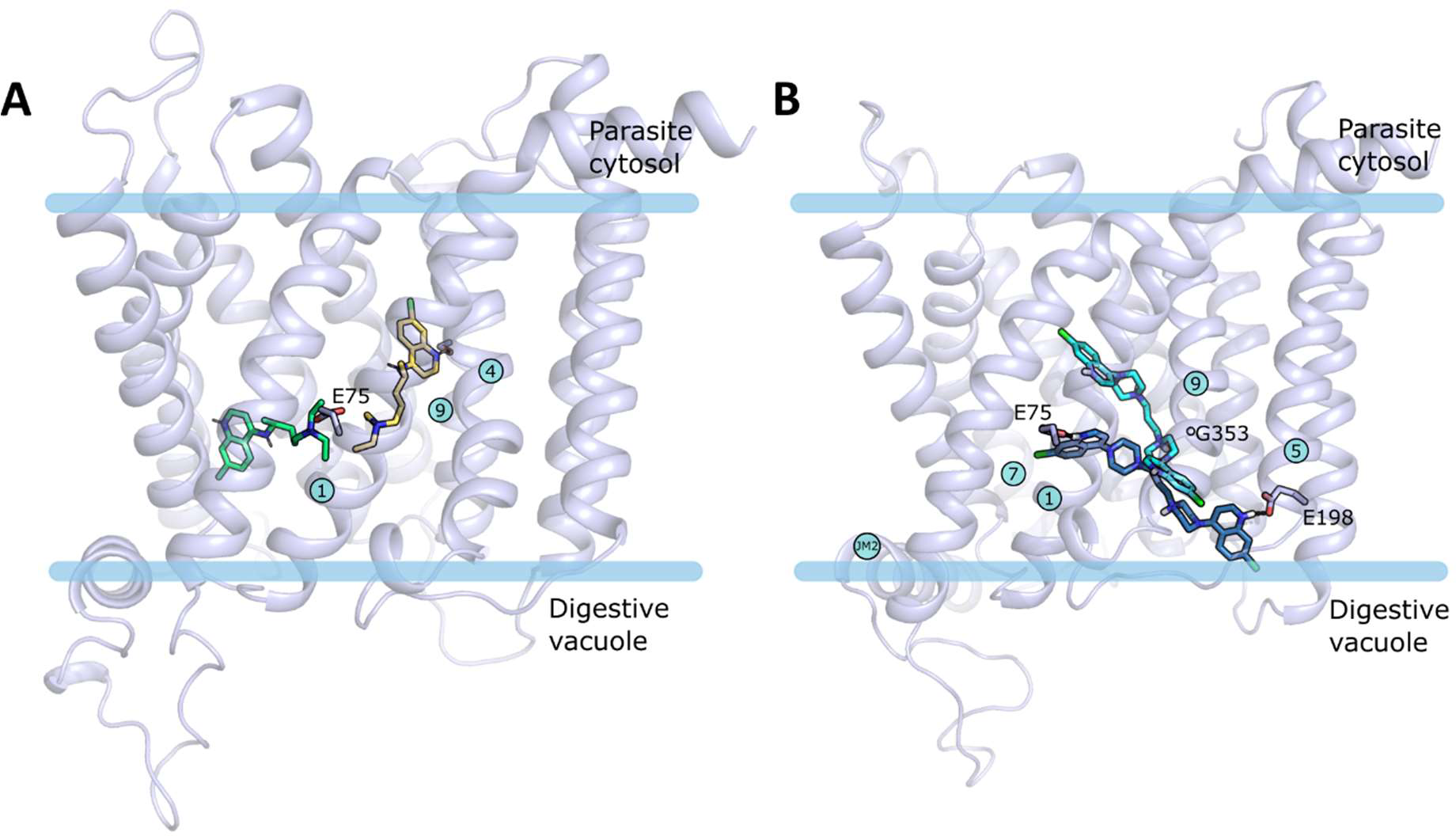

Supplement: S8 Fig — The last frame from the MD simulations is shown and superimposed on the original docking pose for (A) CQ, and (B) PPQ. The docking and the molecular dynamics final poses are shown in green and yellow, respectively, for CQ, and cyan and blue, respectively, for PPQ. The protein and the amino acid residues that interact with ligands are shown in light blue. (TIF) [file ppat.1011436.s011.tif]

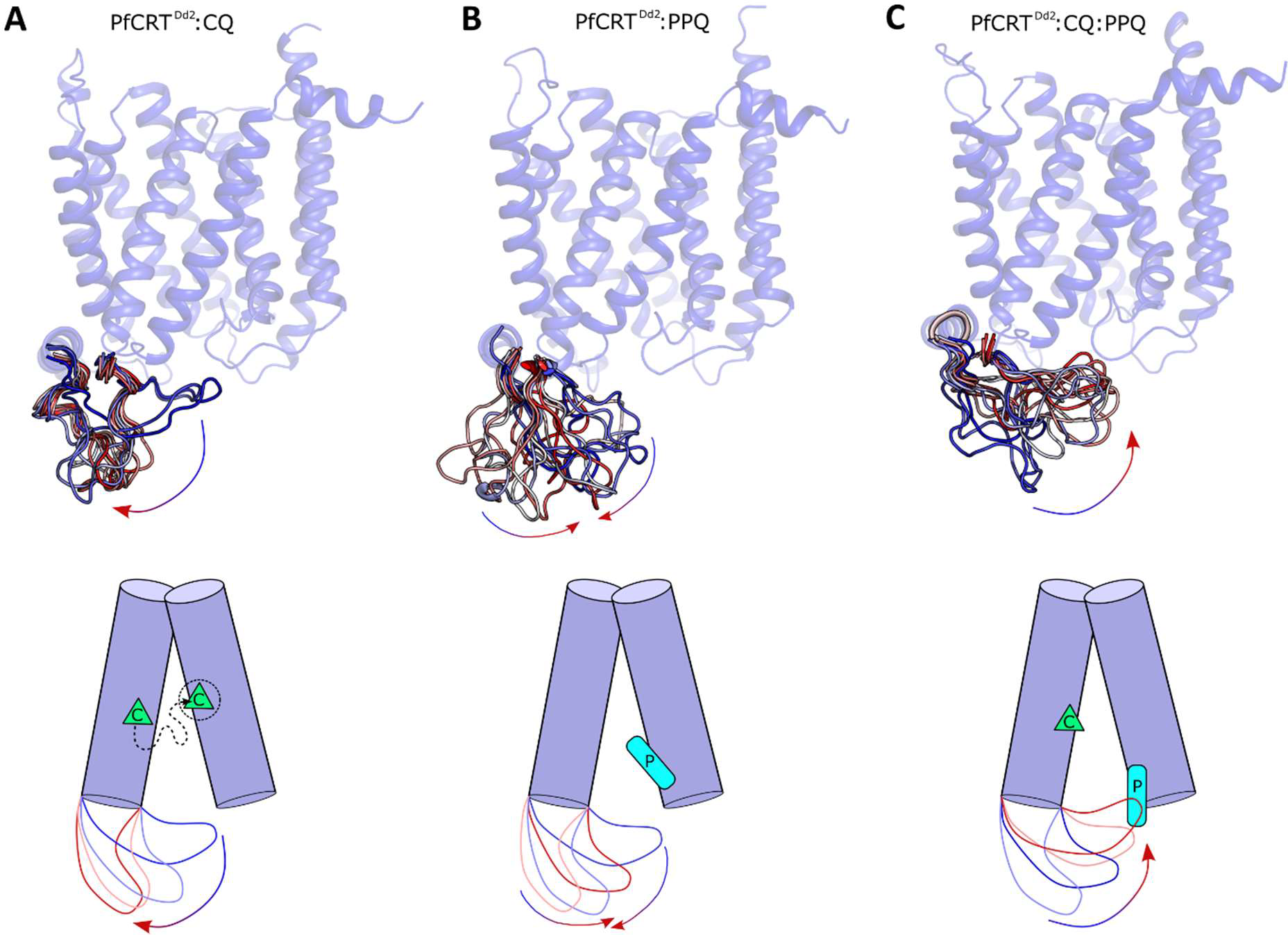

Supplement: S9 Fig — A blue-to-red color scale is used to indicate the movement of the loop along the simulation trajectories, as highlighted by the arrows on the schematic figures. Loop rearrangements are shown for the (A) PfCRTDd2:CQ, (B) PfCRTDd2:PPQ, and (C) PfCRTDd2:CQ:PPQ complexes. Starting and final binding poses (encircled) are shown for CQ in panel (A). (TIF) [file ppat.1011436.s012.tif]

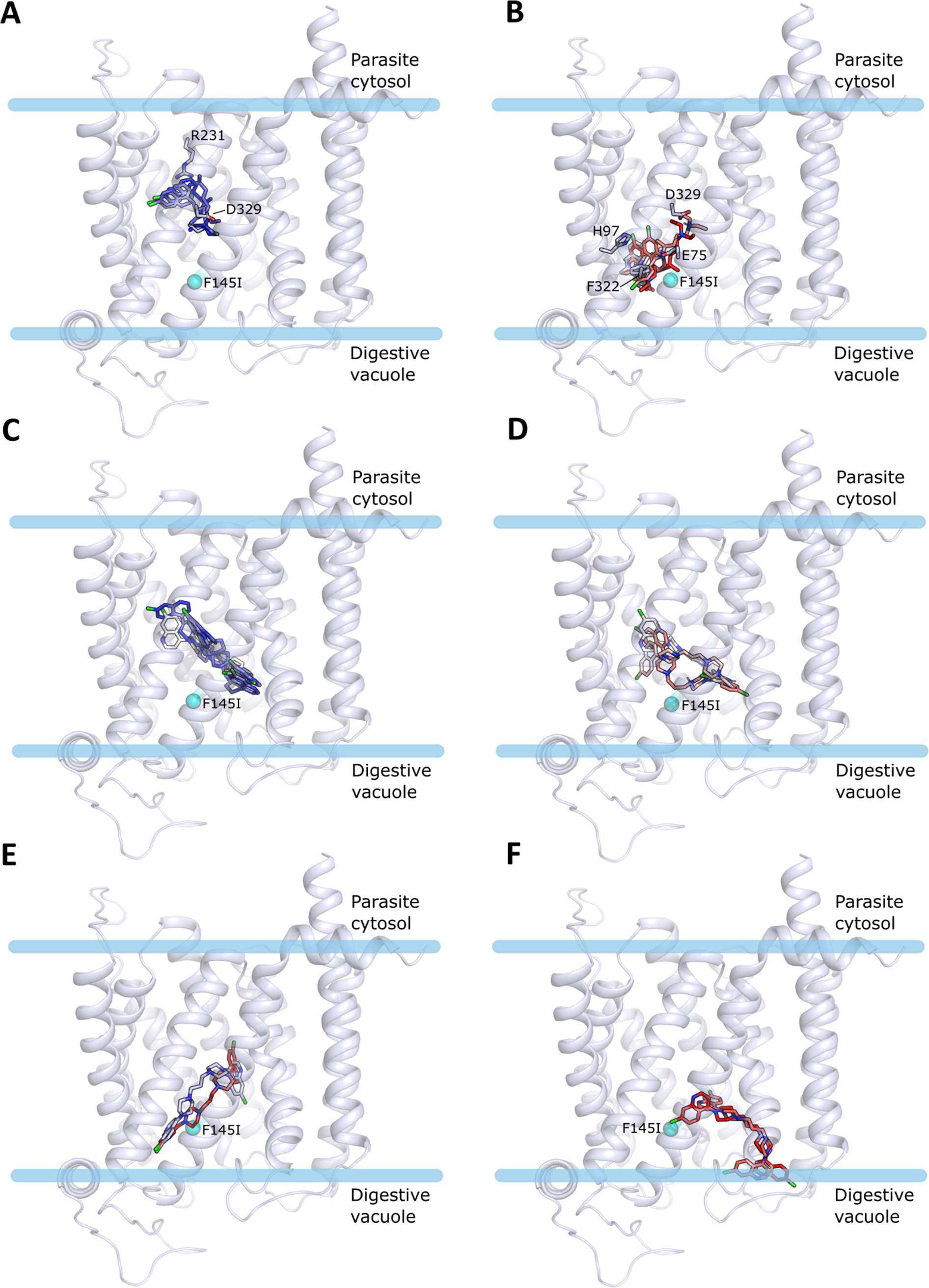

Supplement: S10 Fig — A blue-to-red color scale shows the generated docking poses for CQ (A-B) and PPQ (C-F), ranked from the best (blue) to the worst (red) docking score. The F145I mutation is indicated as a sphere in cyan. (TIF) [file ppat.1011436.s013.tif]

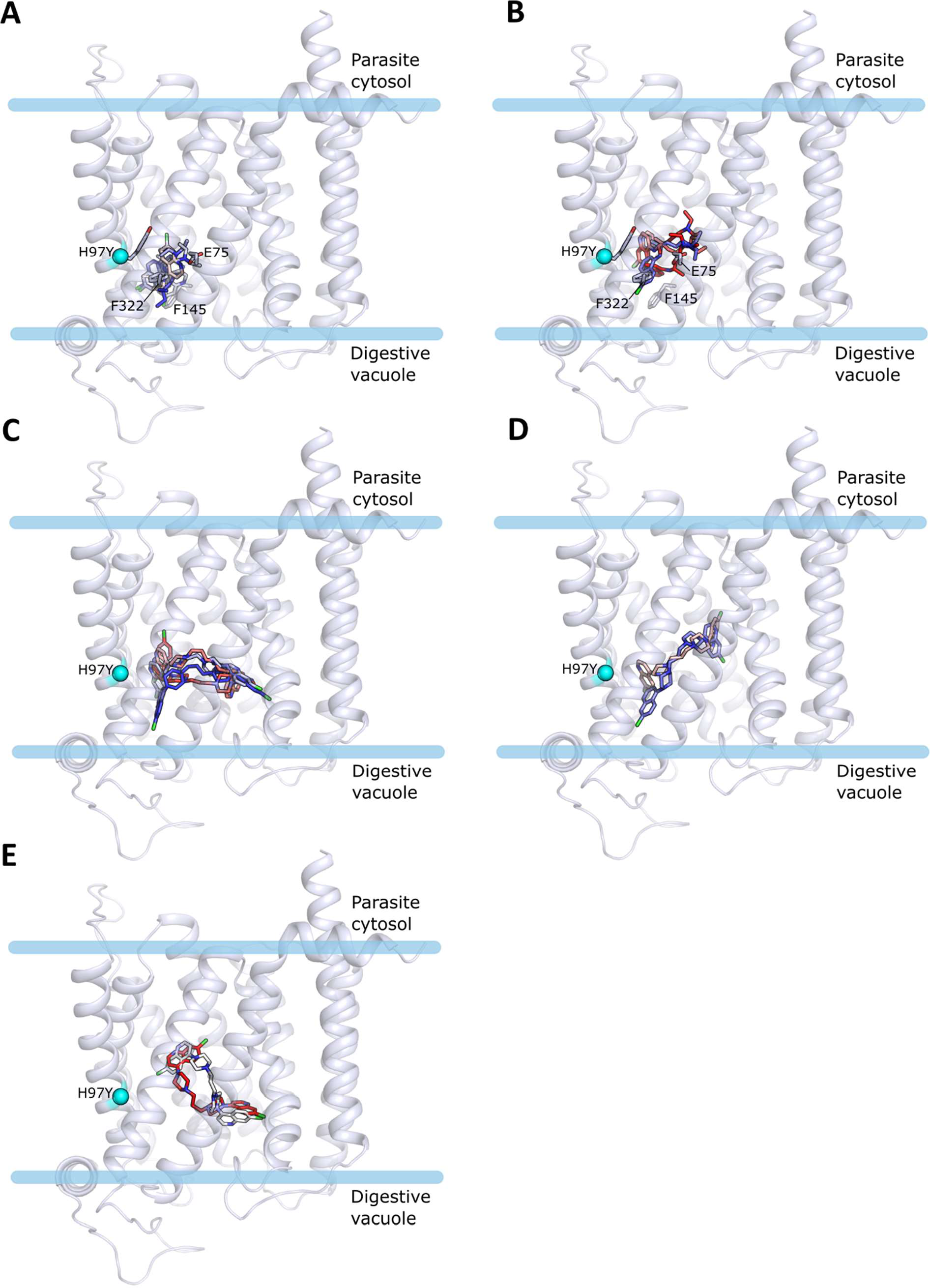

Supplement: S11 Fig — A blue-to-red color scale shows the generated docking poses for CQ (A-B) and PPQ (C-E), ranked from the best (blue) to the worst (red) docking score. The H97Y mutation is indicated as a sphere in cyan. (TIF) [file ppat.1011436.s014.tif]

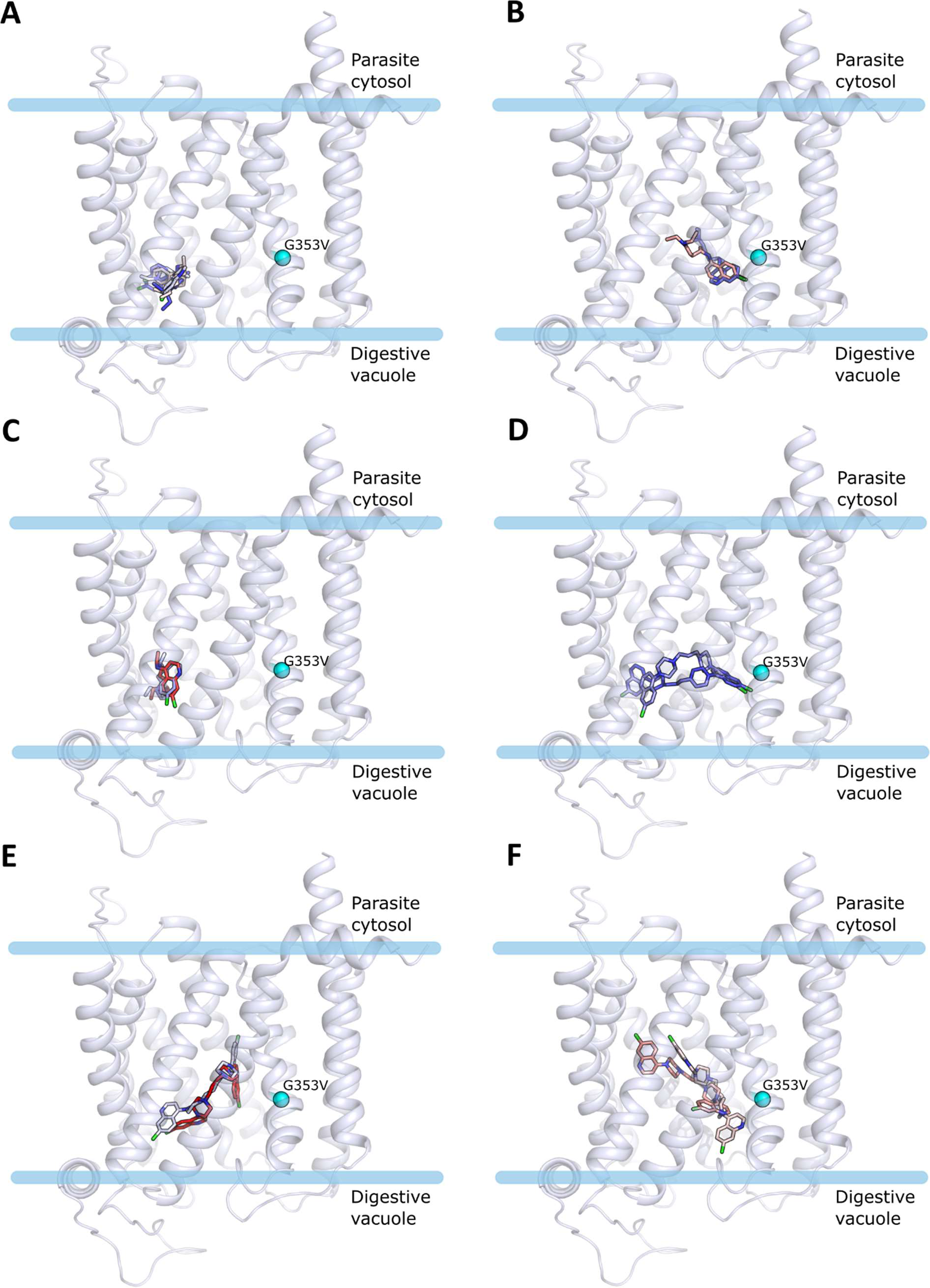

Supplement: S12 Fig — A blue-to-red color scale shows the generated docking poses for CQ (A-C) and PPQ (D-F), ranked from the best (blue) to the worst (red) docking score. The G353V mutation is shown as a sphere in cyan. (TIF) [file ppat.1011436.s015.tif]

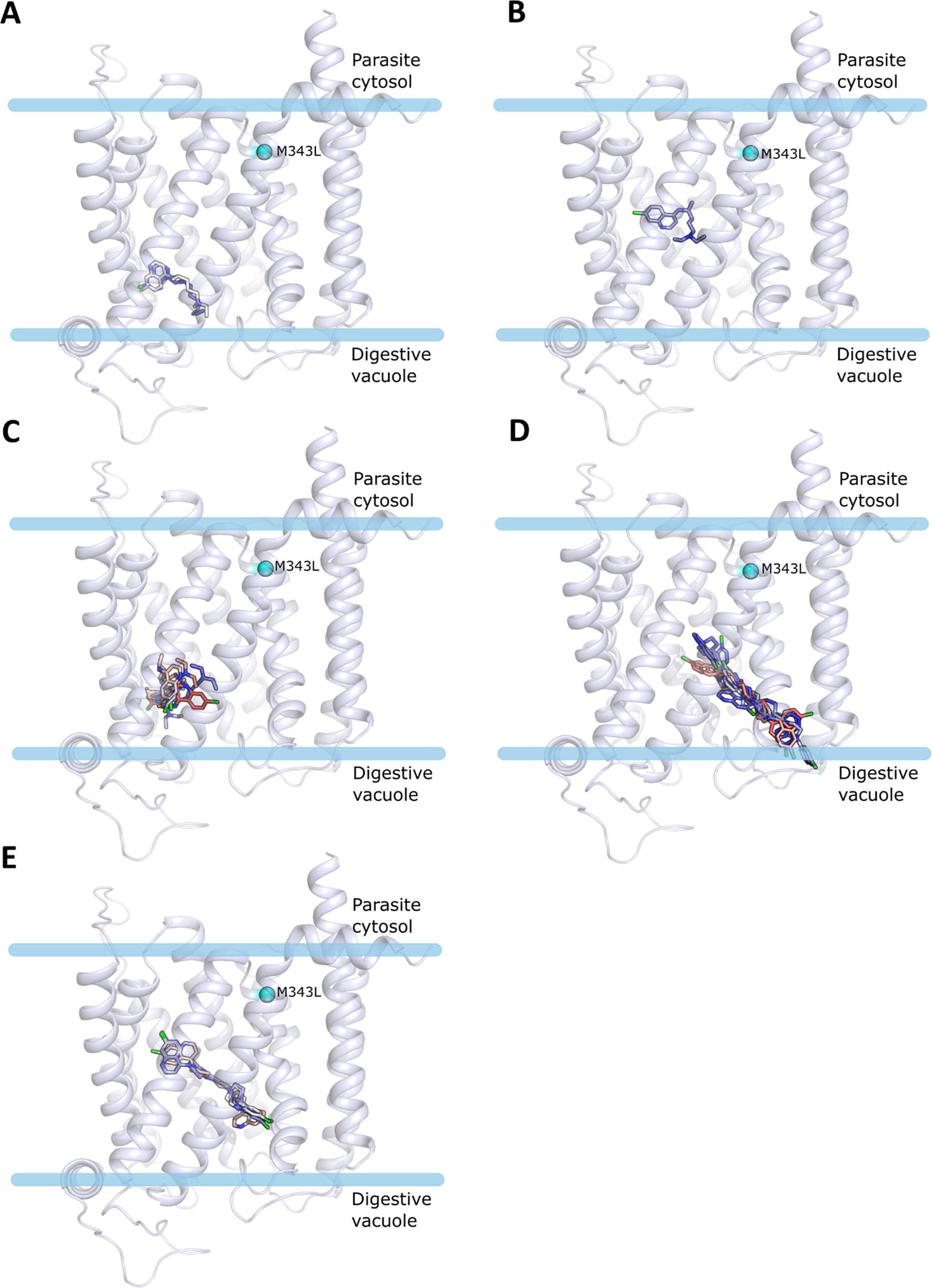

Supplement: S13 Fig — A blue-to-red color scale shows the generated docking poses for CQ (A-C) and PPQ (D-E), ranked from the best (blue) to the worst (red) docking score. The M343L mutation is shown as a sphere in cyan. (TIF) [file ppat.1011436.s016.tif]

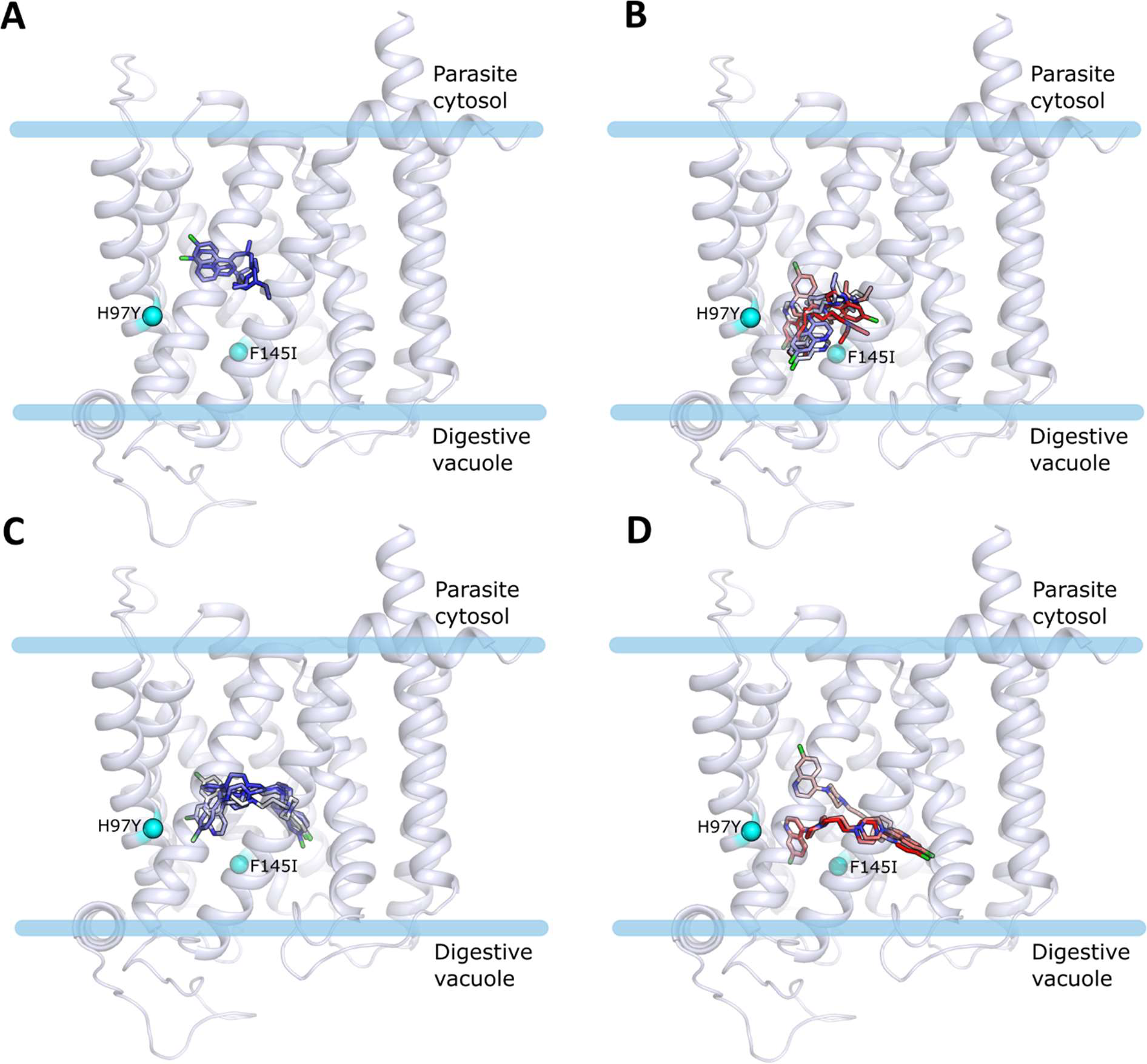

Supplement: S14 Fig — A blue-to-red color scale shows the generated docking poses for CQ (A-B) and PPQ (C-D), ranked from the best (blue) to the worst (red) docking score. The H97Y and F145I mutations are shown as a sphere in cyan. (TIF) [file ppat.1011436.s017.tif]
