# Supplementary material for: PfCRT mutations conferring piperaquine resistance in falciparum malaria shape the kinetics of quinoline drug binding and transport
Source: PLoS Pathog. 2023 Jun 7;19(6):e1011436. doi: 10.1371/journal.ppat.1011436 (PMC10281575; doi:10.1371/journal.ppat.1011436)
Supplement: Attachment — Submitted filename: response to reviewers comments PlosPathogen.docx [file ppat.1011436.s018.docx]

**Responses to reviewers‘ comments on manuscript PPATHOGENS-D-23-00097**

We thank the reviewers for their encouraging comments and helpful suggestions.

**Part I – Summary**

Please use this section to discuss strengths/weaknesses of study, novelty/significance, general execution and scholarship.

Reviewer #1: This study uses the competition kinetics of chloroquine (CQ) and piperaquine (PPQ), together with docking and molecular dynamics simulations, to investigate the mechanism of various mutations in the PfCRT conferring resistances to both drugs. CQ and PPQ competition assays suggest a noncompetitive inhibition mechanism. The results show that PfCRT can accept simultaneously both CQ and PPQ at distinct but allosterically interacting sites. Structural simulation analyses also appeared to support the noncompetitive nature of the two drugs. Additionally, PfCRT from CQ-resistant parasites is better at transporting CQ than are PfCRT variants from PPQ-resistant parasites, and vice versa. These observations provide mechanistic insight into the PfCRT structure and transport functions for the two drugs. Drug resistance in malaria and PfCRT-mediated resistance has been and will continue to be ‘hot topics’ in malaria research. This study will attract the attention of many scientists in the research fields of malaria and other parasites.

One negative part of this study is that the analyses were all performed in X. laevis expression system, not the parasite itself. Studies have shown that some PPQ-resistant mutations in Pf parasites are dependent on the parasite’s genetic background. Maybe the authors can address this point in the Discussion.

We fully agree with the reviewer on the importance of the genetic background in defining PPQ resistance in the parasite. The oocyte system is clearly a reductionistic approach, however, a very useful one when it comes to dissecting a complex phenotype, such as in vivo drug resistance, into different contributing factors. In this study, we solely focused on the contribution of PfCRT and how polymorphisms in this transporter affect the transport kinetics for CQ and PPQ. In response to the reviewer’s comments, we have added the following paragraph to the discussion:

“PPQ resistance is a complex phenotype in the parasite, and although PfCRT is a contributing factor, it is not the only one [8, 27, 36, 37]. The PfCRT variants associated with PPQ resistance often incur a fitness cost due to their altered ability to handle oligopeptides [8, 10, 26], which are the natural substrate of PfCRT [17, 18]. The focus of this study was solely on PfCRT and how mutational changes associated with PPQ resistance affect CQ and PPQ transport kinetics, with the aim of providing a mechanistic understanding of the CQ re-sensitizing phenomenon associated with PfCRT variants contributing to PPQ resistance. Although we took a reductionistic approach by focusing on PfCRT, we believe that our study's conclusions are not limited by this choice.” (discussion, page 18)

Reviewer #2: Mutations in PfCRT play a crucial role in altered drug susceptibility of the malaria parasite to historical antimalarials such as chloroquine (CQ) and more recently to current front-line components of antimalarial treatments such as piperaquine (PPQ). Understanding how PfCRT mutations confer resistance to PPQ may be critical for combatting emerging resistance. Previous studies using the xenopus laevis oocyte system by these authors and others have provided convincing evidence that mutant forms of PfCRT transport CQ with varying kinetics (e.g. PMID: 24728833), and that CQ-resistant PfCRT is able to transport a range of different substrates, likely via interactions at multiple binding sites (PMID: 25378409). Here the authors leverage the same approach and analytical techniques to investigate PfCRT interactions with Piperaquine and chloroquine.

In contrast to prior literature, this study indicates that PPQ is a substrate of pre-existing PfCRT variants (PfCRT-Dd2) which does not itself confer resistance to PPQ. Furthermore, the study indicates that mutations that confer PPQ resistance and a corresponding loss of CQ resistance, retain appreciable levels of CQ transport activity. These findings do not correspond precisely with previous observations in parasites, where correlations between PPQ accumulation and resistance phenotypes are weak. However, the authors make a reasonable argument that nuanced differences in kinetics of drug transport do indeed account for the altered PPQ and CQ susceptibility conferred by these mutations. While the data supporting these claims appear reasonable, additional controls are required to validate the findings, and a more transparent approach to correlating transport with resistance phenotype is required. The analysis also lacks an account of how these mutations influence the physiological role of the transporter, which may itself be an important contribution to the resistance phenotype. It is also puzzling that the transport rate of PPQ reported here is vastly higher than for CQ, and the authors should be sure that all appropriate controls have been included to ensure that only the specific PfCRT-mediated PPQ transport is being detected. The authors also examine the transport properties of an engineered double-mutant (Dd2-H97V-F145I) with unusual kinetic properties, which may or may not confer cross-resistance to both CQ and PPQ. Additional experiments are required to fully substantiate the claims and physiological relevance of this finding.

We fully agree with the reviewer on the importance of appropriate controls and, in response to the reviewer’s comments, have performed additional control experiments to strengthen our findings (see below). We have further improved the correlation analysis and now highlight the direct correlation between PPQ V_max_ values and CQ K_M_ values (revised Fig. 2c). Our study does not inform on the effect PfCRT mutations have on the physiological, non-drug transport function of PfCRT. We are aware of this limitation and fully acknowledge it in the discussion. In the revised manuscript, we now refer to recent publications demonstrating an effect of PfCRT mutations on the oligopeptide handling and transport.

“PPQ resistance is a complex phenotype in the parasite, and although PfCRT is a contributing factor, it is not the only one [8, 27, 36, 37]. The PfCRT variants associated with PPQ resistance often incur a fitness cost due to their altered ability to handle oligopeptides [8, 10, 26], which are the natural substrate of PfCRT [17, 18]. The focus of this study was solely on PfCRT and how mutational changes associated with PPQ resistance affect CQ and PPQ transport kinetics, with the aim of providing a mechanistic understanding of the CQ re-sensitizing phenomenon associated with PfCRT variants contributing to PPQ resistance. Although we took a reductionistic approach by focusing on PfCRT, we believe that our study's conclusions are not limited by this choice.” (discussion, page 18)

Building on previous kinetic studies of CQ and quinine interactions, the authors next examine the inhibition of CQ transport by PPQ, and PPQ transport by CQ in PfCRT-Dd2 expressing oocytes in order to examine the mechanism of PPQ and CQ interactions with PfCRT. This aspect of the work applies appropriate analyses and experiments to establish that PPQ and CQ interact with PfCRT at distinct sites within the substrate binding pocket and influence transport of one another largely by noncompetitive mechanisms. The same mechanisms of interaction were observed in a PPQ resistant form of PfCRT. With the contingent uncertainty around reported PPQ transport rates aside, this aspect of the study is complete and comprehensive. Molecular dynamics simulations and docking studies provide corroborating evidence suggesting plausible sites at which both CQ and PPQ could interact with PfCRT-Dd2. Overall this aspect of the study builds on previous observations by the same group and contribute to the understanding of PfCRT as accommodating a range of substrates via multiple interaction sites.

Overall the manuscript is well written, the overall rational for the study and analysis are sound and treats prior work fairly and appropriately, however additional data would further support the claims made.

Reviewer #3: PfCRT harbors mutations that mediate the efflux of chloroquine (CQ) from the digestive vacuole where the drug action mechanism occurs. Additional mutations on the Southeast Asian CQ-resistant PfCRT Dd2 variant have recently been evolved in response to drug pressure which in turn mediating piperaquine (PPQ) resistance while re-sensitizing CQ. In this manuscript, Gomez et al. have performed transport kinetic studies, competition plot experiments and molecular dynamics simulations to describe how additional mutations associated with PPQ resistance alter transport kinetics of CQ and PPQ, and both drugs can allosterically bind at distinct sites within the drug-binding cavity of PfCRT. Moreover, CQ and PPQ appear to act as partial noncompetitive inhibitors as opposed to competitive inhibitors as previously suggested. The authors have further engineered PfCRT by introducing two mutations associated with PPQ resistance which enhanced transport efficiency for both drugs, suggesting that the rationale of using both CQ and PPQ as a combination therapy may not be the most ideal for malaria treatment.

Understanding how CQ and PPQ bind to PfCRT, and how mutations in PfCRT influences drug transport are significantly important. The findings from this manuscript would provide insight into the molecular mechanism utilized by PfCRT to mediate CQ and PPQ resistance. The manuscript is well-written, and the authors have provided thorough explanations on transport kinetics and competition plot experiments.

I have the following questions and comments:

In the competition plot experiment (Figure 3), I would be inclined to see the competition plot for the interaction of PfCRT Dd2 with CQ/PPQ and Verapamil, a mixed-type inhibitor. In my opinion, this would provide a better understanding on the differences between noncompetitive and mixed-type inhibition models.

The competition plot tests only whether a reaction is competitive (Chevillard et al., 1993). It cannot differentiate between a noncompetitive and a mixed type reaction. The reviewer may elute to the fact that the competition plot shows a concave behavior for CQ/PPQ and a convex behavior for CQ/QN. However, this divergent appearance cannot be interpreted as evidence of a different inhibition type. It is rather the result of the relationship between the Michaelis-Menten constant of a substrate and its inhibition constant for the transport of the second substrate (Chevillard et al., 1993; Cárdenas, 2001).

The authors should comment on their rationale for the use of POPC as a choice of lipid for the simulations.

The lipid composition of the *P. falciparum* membrane is still unknown. POPC is a simple and well-characterized model widely used for membrane simulations and was also the choice for previous molecular dynamics simulations of PfCRT (Kim et al., 2019; Small-Saunders et al., 2022; Willems et al., 2023).

The study reports an intriguing observation on a loop rearrangement in the digestive vacuole upon drug bindings over the simulation time (Fig. 9B and S7 Fig). I’ve always wondered if the loop in question is involved in the gating of substrate/drug through an alternating-access mechanism or if it has other regulatory roles. In my opinion, this phenomenon should be further discussed. Is there perhaps any known transporter with a similar structural feature anchoring substrate and stabilizing the complexes?

We thank the reviewer for the comment and have added the following paragraph to the discussion:

“Furthermore, simulations point to a peculiar role of the vacuolar loop, which is very flexible in the binary complexes (PfCRT^Dd2^:CQ and PfCRT^Dd2^:PPQ), while having a stabilizing effect in the ternary one, altogether providing new hints on the function of the loop which has not been investigated so far. Multiple sequence alignments of different Plasmodium CRT homologs and orthologues show a high degree of conservation in the vacuolar loop, suggesting a mechanistic role [9]. The area of the transporter facing the digestive vacuole, near the vacuolar loops, was proposed as the initial binding site for CQ [42], thus supporting a possible role of the loop in the initial phase of substrate binding. Several membrane transporters are known to have extracellular loops acting as gates and taking part in the whole transport mechanisms [43], thus supporting the idea that such behavior could hold true for PfCRT.” (discussion, page 21).

The term ‘extracellular loop’ may be confusing, as it is on the digestive vacuole side. I suggest the authors use a different term or specify the location of loop between TM 7 and 8.

We have modified the term ‘extracellular loop’ with ‘vacuolar loop’.

The docking was performed on a homology model. Have the authors tried docking/simulation for CQ and PPQ on the cryo-EM structure of the PfCRT 7G8 isoform? I think a comparison between the two isoforms could further support the locations where CQ and PPQ bind in the PfCRT CQ-resistant isoforms and enhance robustness of the Dd2 homology models.

We thank the reviewer for the suggestion. We have performed docking of CQ and PPQ on the cryo-EM structure of the PfCRT^7G8^ isoform and observed a good agreement between the docking of CQ in the two isoforms, with the first two poses located in the top central part of the cavity and all the remaining ones located in the vicinity of F145. Conversely, PPQ mainly binds to the center-left side of the cavity with a quite conserved pose that interacts with F145 via one aminoquinoline group and with S349 via the other, without poses uniquely occupying the right side of the channel and resembling those detected in the Dd2 isoform.

We have inserted a comment on this in the Results and Discussion section:

“In a previous study, it was proposed that PPQ and CQ act as competitive inhibitors of each other on PfCRT^7G8^. To explore a possible isoform-specific binding behavior, we conducted a docking analysis using PfCRT^7G8^. While the docking of CQ to PfCRT^7G8^ agreed with that of PfCRT^Dd2^, PPQ mainly occupied the center-left side of the cavity without entirely occupying the right side (Fig 10A-E). The amino acid sequence differences on the right side of the transporter (I356 and R371 in PfCRT7G8 and T356 and I371 in PfCRTDd2) (Fig 10F) may impede PPQ accommodation in the 7G8 isoform, leading to overlapping binding sites for CQ and PPQ. These results support the suggested competitive binding of the drugs to PfCRT^7G8^ [9], and suggest a noncompetitive binding model specific to the PfCRT^Dd2^ isoform.” (results, page 15).

“Interestingly, a comparison with the dockings of both drugs to PfCRT^7G8^ suggests an isoform-specific behavior. While the docking of CQ to PfCRT^7G8^ is pretty much in agreement with that to PfCRT^Dd2^, PPQ mainly binds to the center-left side of the cavity with no poses entirely occupying the right side (Fig 10A-E). The differences in the amino acid sequence of the right side of the transporter (I356 and R371 in PfCRT^7G8^ and T356 and I371 in PfCRT^Dd2^) (Fig 10F) could hamper the accommodation of PPQ in the 7G8 isoform, resulting in overlapping binding sites for CQ and PPQ. These findings are in agreement with the proposed competitive binding of the two drugs to PfCRT^7G8^ [9] and let us hypothesize that a noncompetitive binding model is specific for the PfCRT^Dd2^ isoform.” (discussion, page 20).

**Part II – Major Issues: Key Experiments Required for Acceptance**
Please use this section to detail the key new experiments or modifications of existing experiments that should be absolutely required to validate study conclusions.

Generally, there should be no more than 3 such required experiments or major modifications for a "Major Revision" recommendation. If more than 3 experiments are necessary to validate the study conclusions, then you are encouraged to recommend "Reject".

Reviewer #1: (No Response)

Reviewer #2: Figure 1 shows that PPQ transport rates were consistently higher than for CQ. Given that PfCRT-Dd2 bearing parasites have no difference in PPQ accumulation compared to CQ sensitive PfCRT-3D7 parasites (e.g. PMID: 36306287), how do the authors explain this apparent high rate of PPQ transport? Were the figures in panel A and B conducted within oocytes from the same batch? The authors should include a wild-type PfCRT control (or another unrelated transporter not involved in drug transport) in Figure 1 that would not be expected to transport CQ or PPQ for additional validation. The relatively small difference in drug uptake between water-injected and PfCRT-expressing oocytes compared to previous studies (e.g. PMID:25378409) is concerning, and suggests a low signal to noise level that would reduce the sensitivity of subsequent kinetics. The findings of high levels of PPQ transport via PfCRT-Dd2 also contradict recent observations indicating that Dd2 PfCRT is unable to transport PPQ (PMID: 35130315). Given these discrepancies the authors should seek corroborating evidence that the rates of PPQ transported reported here are due entirely to specific transport of the drug by PfCRT-Dd2.

We fully agree with the reviewer on the importance of control experiments and, in response to the reviewer’s comment, now include PPQ and CQ uptake kinetic data for the wild type PfCRT^HB3^ variant. As seen in the revised Figure 1A, the time courses of CQ and PPQ uptake by PfCRT^HB3^-expressing oocytes is not different from those of water-injected oocytes, thus, confirming the background level. Throughout the manuscript, we carefully differentiate between drug accumulation, which refers to the total amount of drug taken up by the oocyte, and PfCRT-mediated uptake, which indicates the amount of drug associated with the PfCRT specific transport activity. The latter was calculated by subtracting the amount of drug taken up by water-injected oocytes by that of PfCRT-expressing oocytes. We would further like to emphasize that all our experiments are controlled by investigating PfCRT^Dd2^-expressing oocytes and water-injected oocytes in parallel experiments. The specific PfCRT-mediated drug transport activity is, therefore, calculated in each experiment. An experiment is defined by oocytes prepared from a particular frog at a particular day. As an additional control, we use oocytes from at least three different frogs per condition – of course in independent experiments and not at the same day. As a third control, we demonstrated that PfCRT-mediated CQ and PPQ uptake is sensitive to verapamil (S3 Fig).

On other points raised by the reviewer, we beg to disagree. We have performed PfCRT-mediated transport kinetics in the oocyte system by several different PhD students over many years and found that the assay is very robust and has produced highly reproducible data. For example, the data on PfCRT^Dd2^-mediated CQ transport presented in the current study are comparable to those shown by Bellanca et al., and even correspond to those published by Rowena Martin and her team. To illustrate this point, we list below the apparent Km for CQ on PfCRT^Dd2^: 260 ± 10 (this study); 250 ± 30 (Bellanca et al., 2014); 245 ± 3 (Martin et al. 2009).

We assume the reviewer refers to Figure 1 in Okombo et al. (2022) in his/her comment on equal amounts of CQ and PPQ accumulation by the PfCRT^Dd2^ and PfCRT^3D7^ carrying parasites. However, we are not sure if we understood the reviewer correctly. The aforementioned Figure 1 shows that the parasite strain Dd2^Dd2crt^ and the Dd2^3D7crt^ accumulate PPQ 1900- and 2000-fold, respectively. In the case of CQ, the accumulation ratios are ~25- and 1000-fold, respectively. No matter how you twist and turn it, PPQ accumulation ratios always exceed those of CQ.

The study by Small-Saunders et al. reported CQ uptake by both PfCRT^Dd2^ and PfCRT^Dd2+F145I^, yet with different kinetics. This finding is fully consistent with our data. Similarly, both studies agree on PfCRT^Dd2+F145I^ transporting PPQ. Where the two studies diverge is the response of PfCRT^Dd2^ to PPQ. We observed a specific, verapamil-sensitive PPQ transport activity, whereas Small-Saunders et al. did not. It is difficult to compare and evaluate both studies from a distance. However, we noted that the aforementioned study used PPQ concentrations in the time course (75nM) and the kinetic analysis (1-4 µM) that were 2 to 3 orders of magnitude below the K_M_ of PfCRT^Dd2^ for PPQ (125 µM as determined in our study). It is, therefore, possible that PPQ transport by PfCRT^Dd2^ was not detected by Small-Saunders et al. because of a very low substrate concentration. In comparison, we used PPQ concentrations of 50 µM for the time course experiment and 10-500 µM for the kinetic analysis. Please don’t misunderstand us. We do not wish to criticize the aforementioned study. Quite the contrary, we respect and appreciate the work. However, the evidence against a PPQ transport activity by PfCRT^Dd2^ is not as strong as it first appears (see also the negative transport activity in the time course). We further would like to point out that an unrelated study by Riegel and Roepe presented evidence of PfCRT^Dd2^-mediated PPQ transport in a yeast expression system (Riegel and Roepe, 2020).

In response to the reviewer’s comment, we now state in the discussion that PfCRT^Dd2^-mediated PPQ transport has been “shown by some but not all studies”. (discussion, page 18).

The authors assert that relatively subtle differences in drug transport kinetics explain differing levels of CQ and PPQ resistance in parasites bearing existing and novel PfCRT mutations. This conclusion relies largely on a Principal Component Analysis of transport data for five PfCRT variants along with corresponding drug-resistance data from other sources. Critical controls have not been conducted (i.e. expression levels for all variants tested by western blot and inclusion of a bona fide CQ and PPQ-sensitive PfCRT variant), and the PSA plot is unclear (see part III below). The findings also contradict recent observations indicating that transport alone cannot account for the resistance phenotype of these mutations in isogenic parasites (PMID: 36306287) and no discussion of the impact of these mutations on the physiological role of PfCRT is attempted.

We thank the reviewer for the suggestions. As mentioned above, we now include data on CQ and PPQ transport by PfCRT^HB3^, which we found not to be significantly different from that of water-injected oocytes (see also Martin et al., 2009 for CQ transport).

We concur with the reviewer's comments on the PSA. We acknowledge that the in vivo drug response values reported by different groups vary (Ross et al., 2018; Okombo et al., 2022), and our study does not shed any new light on this issue. Therefore, we replaced the PSA by a correlation analysis, comparing the PPQ V_max_ and CQ K_M_ value for each variant. The revised Figure 2c now shows a direct correlation between these two kinetic parameters.

We also now show, by IFA and semi-quantitative Western analysis, comparable expression levels for the most relevant PfCRT variants investigated in this study (S1 and S2 Fig). Furthermore, we now mention the cited work and discuss the impact these mutations have on parasite fitness and oligopeptide handling. In addition, we include a paragraph on the limitations of our work.

“PPQ resistance is a complex phenotype in the parasite, and although PfCRT is a contributing factor, it is not the only one [8, 27, 36, 37]. The PfCRT variants associated with PPQ resistance often incur a fitness cost due to their altered ability to handle oligopeptides [8, 10, 26], which are the natural substrate of PfCRT [17, 18]. The focus of this study was solely on PfCRT and how mutational changes associated with PPQ resistance affect CQ and PPQ transport kinetics, with the aim of providing a mechanistic understanding of the CQ re-sensitizing phenomenon associated with PfCRT variants contributing to PPQ resistance. Although we took a reductionistic approach by focusing on PfCRT, we believe that our study's conclusions are not limited by this choice.” (discussion, page 18).

The transport properties of a double-mutant (PfCRT-Dd2-H97Y-F145I) could render both CQ and PPQ resistance, however at many concentrations this variant has lower drug transport rates than other variants tested. Were CQ and PPQ kinetics experiments conducted in parallel with PfCRT Dd2-expressing oocytes, or is the dotted line in figure 10 data from other experiments? Ideally these comparisons would be made in pairwise experiments with oocytes from the same batch. The expression levels of this variant were also not assessed. Both the H97Y and F145I mutations incur high fitness costs in multiple PfCRT and genome backgrounds of the parasite (PMID: 30115924, 36082431 36306287, 35130315), suggesting that a double mutant would be exceedingly unlikely to be viable. If the authors wish to make this a key finding of the study, efforts should be made to test the contribution of this variant to altered drug susceptibility in parasites (e.g. via episomal expression as previously conducted by this group PMID: 24728833).

We appreciate the reviewer's feedback. It is important to note that the double mutant resulting from our comparative kinetic analyses is not the primary focus of our study. Nonetheless, combining the H97Y and F145I mutations seemed like an obvious next step in our work, and had we not tested it, the reviewers would have likely asked for it. We do acknowledge in the discussion that we did not test the double mutant in the parasite, and therefore, have no information on its viability. However, even if the mutant is not viable, it may not necessarily imply that PfCRT is incapable of overcoming the re-sensitization effect. It is possible that parasite viability could be restored by compensatory mutations in PfCRT or other factors.

“However, further experiments in the parasite are needed to investigate whether such a double mutant is viable and confers a selective advantage in the presence of CQ and PPQ.” (discussion, page 22).

Reviewer #3: The molecular dynamics simulations and docking studies have demonstrated that CQ likely binds to a region that includes TM 1, 2, 3 and 7 via a salt bridge with E75, a hydrogen bond with S257 and hydrophobic contacts with I260, V141 and F145. Given the rather poor docking score for CQ, I think that characterizing both binding and transport properties of these residues following mutagenesis could increase the confidence of the molecular docking results.

We thank the reviewer for this insightful comment. We have generated and analyzed the three mentioned mutants and found that all three of them display a reduced CQ transport activity (S6 Fig). The additional data are now mentioned in the revised results section:

“Substituting alanine for each of V141, S257, I260 significantly reduced PfCRT-mediated CQ transport in all three cases, as compared with PfCRT^Dd2^ (p<0.001; ANOVA test) (S6 Fig.).” (results, page 13).

**Part III – Minor Issues: Editorial and Data Presentation Modifications**
Please use this section for editorial suggestions as well as relatively minor modifications of existing data that would enhance clarity.

Reviewer #1: A PfCRTDd2_H97Y_F145I double mutant was also generated and tested for responses to CQ and PPQ. The curves showed a sigmoidal response, reflecting the different impacts of the two mutations on the drugs. Is it possible that at lower drug concentrations, the H97Y contributed more to the changed transport activity, whereas at higher concentrations, the F145I was the major player?

We thank the reviewer for this comment. It is a possible explanation. However, we have no data to support it.

Isoborogram is a widely used method for evaluating drug interactions. Did the authors try this method?

We thank the reviewer for this comment. Yes, we have tried isobolograms, but found them to be inconclusive because the responsiveness of the parasite to these two drugs is assessed in different assays – growth inhibition assay and IC_50_ determination in the case of CQ and parasite survival assay in the case of PPQ (Ross et al., 2018).

Line 132, ‘significant’ more CQ’. But there is no significant test.

We apologize for the oversight and have added now information regarding the statistical test used (results, page 7). We have added the following sentences to the results section:

“Oocytes expressing PfCRT^Dd2^ or PfCRT^Dd2_F145I^ took up significantly more CQ and PPQ than did water-injected and PfCRT^HB3^-expressing control oocytes (Figs 1A and 1B, left panels) (PfCRT^Dd2^: *p* <0.001, df = 8, F = 74.59; *p* <0.05, df = 8, F = 4.61; for CQ and PPQ, respectively; PfCRT^Dd2_F145I^: *p* <0.001, df = 8, F = 26.58; *p* <0.005, df = 8, F = 15.17; for CQ and PPQ, respectively; F-test).” (results, page 7)

Figures 2A and 2B are not cited in the text.

We apologize for the oversight and now mention Figure 2A and B in the text (results, page 8).

Figure 2C legend, please explain the arrows and the why the specific parameters used in the PCA. Why and how IC50 for CQ, and PSA and CAR for PPQ were chosen for the analysis?

As also suggested by reviewer 2, we have replaced the PCA by a correlation analysis. We acknowledge that the in vivo drug response values reported by different groups vary (Ross et al., 2018; Okombo et al., 2022), and our study does not shed any new light on this issue. Therefore, we replaced the PSA by a correlation analysis, comparing the PPQ V_max_ and CQ K_M_ value for each variant. The revised Figure 2c now shows a direct correlation between these two kinetic parameters.

Fig. S4. Can the different poses be displayed in sequential figures? It is difficult to see the changes in the mixed poses in the same figure.

We understand the reviewer’s comment. Our aim was to give a general view of the areas explored by the two drugs during docking. We have modified S4 Fig (and made accordingly Figures S9 to S13) by showing in different panels the most representative clusters of poses for CQ and PPQ in the different PfCRT variants.

Fig. S5, spell out RMSD.

We have modified the figure’s caption accordingly.

Reviewer #2: Table 1 and subsequent tables should include the number of replicates for each construct/substrate in addition to mean ± SEM.

We thank the reviewer for this suggestion and have included the requested information in the Tables.

The PSA plot and analysis in Figure 2 is not a sufficiently clear illustration of the relationships between resistance and drug transport activity. No grouping of PPQ resistant variants is evident, and it is not clear whether the vectors displayed are scaled by weighting or significance. PSAs on small datasets can also be misleading by obscuring outlier values that may drive overall trends and can overstate the contribution of variables with small ranges. If the authors wish to establish a correlation between measures of in vitro drug susceptibility and drug transport properties such as Vmax or Km, (or a composite metric such as Vmax/Km) for various PfCRT variants, it would be preferable to present these correlations directly. The cellular resistance data for H97Y is taken from a strain with a different genetic background (PH1263-C) to the other variants (Dd2) and may confound the analysis. More up to date data including PPQ and CQ cellular accumulation ratios published in Okombo & Mok et al 2022 should also be included (PMID: 36306287).

We concur with the reviewer's comments on the PSA. We acknowledge that the in vivo drug response values reported by different groups vary (Ross et al., 2018; Okombo et al., 2022), and our study does not shed any new light on this issue. Therefore, we replaced the PSA by a correlation analysis, comparing the PPQ V_max_ and CQ K_M_ value for each variant. The revised Figure 2c now shows a direct correlation between these two kinetic parameters.

Supplementary figure S1 panel A could include a merge of WGA, PfCRT and DIC to illustrate that PfCRT expression is indeed at the surface of the oocytes.
Supplementary figure S1B indicates large discrepancies in expression levels in oocytes from different batches, and some experiments resulted in markedly higher expression levels of one genotype compared to another. What efforts were made to control for expression levels in drug transport experiments comparing different PfCRT variants? Were transport rates normalized to internal controls? How were differing expression levels accounted for between biological replicates? If any normalizations were performed, the authors should clearly state so in the methods and figure legends.

We thank the reviewer for this suggestion and now present IFA and semi quantitative Western analysis data for PfCRT^Dd2^, PfCRT^HB3^, the three PPQ associated PfCRT variants and the double mutant (new S1 and S2 Fig). These data indicated that all PfCRT variants are present on the oolemma and are expressed at comparable levels, according to an ANOVA test. PfCRT expression levels were normalized to the internal control α-tubulin. As indicated in the manuscript, we did not normalize transport rates. Instead, we show absolute mean values form a sufficiently high number of biological replicates (at least four biological replicates as defined by oocytes from different frogs, with 10 oocytes per condition).

It is unclear why certain docking representations were included in Figure 6 – was this due to proximity to ‘interesting’ residues, or due to calculated affinity? Are the same binding modes for PPQ and CQ present in PPQ resistant PfCRT variants? How do the mutations impact docked affinity? How do the identified CQ and PPQ binding sites compare with other recent PfCRT modelling studies (i.e. PMID: 36800498)? Molecular docking with the H97H-F145I double-mutant could also shed light on the possibility of cooperativity in this transporter.

We thank the reviewer for the comments. The docking poses shown in Fig 6 are the ones that were then submitted to Molecular Dynamics simulation. For this purpose, we took the best poses that bind in the two putative sites: the top-ranked pose for PPQ (on the right side of the channel) and the best-scored pose from the cluster around F145 (i.e., the third pose) for CQ.

We have performed additional dockings on the PfCRT mutants under study and added a corresponding paragraph ‘’Docking simulations to the PPQ-resistant PfCRT^Dd2^ variants” in the Result section (Page 15 and following), implemented S1 Table with the additional docking scores, and added Figures S10 to S13 with the corresponding docking poses for each variant. We have also included comments on the different mutants throughout the discussion section.

We have also performed docking to the double mutant (Figure S14) which still shows the ability of CQ and PPQ to bind, largely in agreement with the observation retrieved from the other dockings, supporting the region around F145 as primary for CQ, while having PPQ mostly filling the whole cavity. However, to have insights into the cooperativity of the system, extensive MD simulations would be needed.

In Willems et al. (2023), the authors propose an initial binding site for CQ, which, during MD, evolves into site B, a region that encompasses the central-top area of the transporter and that is comparable to the one we have observed in our calculations. They identify the interaction with D329, and the nearby residues, as key for the transportation of the drug. Their findings align well with our observations: we observed the interaction with D329 in many docking poses both in the 7G8 and Dd2 isoform and transiently during MD simulation of the Dd2 isoform. The work by Willems et al. is now cited in the manuscript:

“The area of the transporter facing the digestive vacuole, near the vacuolar loops, was proposed as the initial binding site for CQ [42], thus supporting a possible role of the loop in the initial phase of substrate binding.” (discussion, page 21).

Reviewer #3: The legends for Figure 8-10 need to be correctly labeled.

We apologize for this oversight and have corrected the mistake.

Figure 2A, B needs to be cited in the text.

We apologize for the oversight and now cite Figure 2B in the text.

The full name for ‘PC’ on the axis in Figure 2C should be indicated in the figure legend.

The PCA was replaced by a correlation analysis as suggested by this reviewer and reviewer 2 (see above).

In all Figure legends, authors used abbreviations for CQ and PPQ but not in Figure 9. I advocate for consistency.

We have now used the abbreviations also in Figure 9.

In Figure 9, the molecular dynamics poses for CQ and PPQ should be colored differently.

We have changed the color for the MD poses as requested for Figure 9, adapting accordingly also Figure S8.

Additional references

1.	Chevillard C, Cárdenas ML, Cornish-Bowden A. The competition plot: a simple test of whether two reactions occur at the same active site. Biochem J. 1993;289 ( Pt 2)(Pt 2):599-604. https://doi.org/10.1042/bj2890599 PMID: 8424801.

2.	Cárdenas ML. The competition plot: A kinetic method to assess whether an enzyme that catalyzes multiple reactions does so at a unique site. Methods. 2001;24(2):175-180. https://doi.org/10.1006/meth.2001.1178 PMID: 11384192.

3.	Kim J, Tan YZ, Wicht KJ, Erramilli SK, Dhingra SK, Okombo J, et al. Structure and drug resistance of the Plasmodium falciparum transporter PfCRT. Nature. 2019;576(7786):315-320. https://doi.org/10.1038/s41586-019-1795-x PMID: 31776516.

4.	Small-Saunders JL, Hagenah LM, Wicht KJ, Dhingra SK, Deni I, Kim J, et al. Evidence for the early emergence of piperaquine-resistant Plasmodium falciparum malaria and modeling strategies to mitigate resistance. PLoS Pathog. 2022;18(2):e1010278. https://doi.org/10.1371/journal.ppat.1010278 PMID: 35130315.

5.	Willems A, Kalaw A, Ecer A, Kotwal A, Roepe LD, Roepe PD. Structures of Plasmodium falciparum Chloroquine Resistance Transporter (PfCRT) isoforms and their interactions with chloroquine. Biochemistry. 2023;62(5):1093-1110. https://doi.org/10.1021/acs.biochem.2c00669 PMID: 36800498.

6.	Okombo J, Mok S, Qahash T, Yeo T, Bath J, Orchard LM, et al. Piperaquine-resistant PfCRT mutations differentially impact drug transport, hemoglobin catabolism and parasite physiology in Plasmodium falciparum asexual blood stages. PLoS Pathog. 2022;18(10):e1010926. https://doi.org/10.1371/journal.ppat.1010926 PMID: 36306287.

7.	Bellanca S, Summers RL, Meyrath M, Dave A, Nash MN, Dittmer M, et al. Multiple drugs compete for transport via the Plasmodium falciparum chloroquine resistance transporter at distinct but interdependent sites. J Biol Chem. 2014;289(52):36336-36351. https://doi.org/10.1074/jbc.M114.614206 PMID: 25378409.

8.	Martin RE, Kirk K. The malaria parasite's chloroquine resistance transporter is a member of the drug/metabolite transporter superfamily. Mol Biol Evol. 2004;21(10):1938-1949. https://doi.org/10.1093/molbev/msh205 PMID: 15240840.

9.	Riegel B, Roepe PD. Altered drug transport by Plasmodium falciparum chloroquine resistance transporter isoforms harboring mutations associated with piperaquine resistance. Biochem. 2020;59(27):2484-2493. https://doi.org/10.1021/acs.biochem.0c00247 PMID: 32589406.

10.	Ross LS, Dhingra SK, Mok S, Yeo T, Wicht KJ, Kümpornsin K, et al. Emerging Southeast Asian PfCRT mutations confer Plasmodium falciparum resistance to the first-line antimalarial piperaquine. Nat Commun. 2018;9(1):3314. https://doi.org/10.1038/s41467-018-05652-0 PMID: 30115924.
